# Supplementary material for: Intracellular antibody signalling is regulated by phosphorylation of the Fc receptor TRIM21
Source: eLife. 2018 Apr 18;7:e32660. doi: 10.7554/eLife.32660 (PMC5906095; doi:10.7554/eLife.32660)
Supplement: Supplementary file 1. [file elife-32660-supp1.docx]

**Data collection and refinement statistics**

|  | 5OLM |
| --- | --- |
| **Data collection** |  |
| Space group | P22121 |
| Cell dimensions |  |
| *a*, *b*, *c* (Å) | 28.4, 83.3, 117.6 |
| α, β, γ (°) | 90.0, 90.0, 90.0 |
| Resolution (Å) | 67.95-1.95 (2.00-1.95) |
| *R*_meas_ | 8.4 (98.6) |
| CC_1/2_ (%) | 99.9 (85.6) |
| CC_1/2anom_ | 42.8 (7.6) |
| *I* / σ*I* | 15.9 (2.2) |
| Completeness (%) | 98.9 (99.4) |
| Redundancy | 7.3 (7.5) |
|  |  |
| Resolution (Å) | 2.0 |
| No. reflections | 21198 |
| *R*_work_ / *R*_free_ | 0.21/0.26 |
| No. atoms | 11253 |
| Protein | 1995 |
| Ligand/ion | 8 |
| Water | 37 |
| *B*-factors |  |
| Protein | 41.5 |
| Ligand/ion | 36.3 |
| Water | 34.2 |
| R.m.s. deviations |  |
| Bond lengths (Å) | 0.018 |
| Bond angles (°) | 1.9 |

*Values in parentheses are for highest-resolution shell.
